# Evaluation of Repellent Effectiveness of Polyvinyl Alcohol/*Eucalyptus globules* Nanofibrous Membranes against *Forcipomyia taiwana*

**DOI:** 10.3390/polym12040870

**Published:** 2020-04-10

**Authors:** Ching-Wen Lou, Ming-Chun Hsieh, Chao-Tsang Lu, Mei-Feng Lai, Mong-Chuan Lee, Bing-Chiuan Shiu, Jia-Horng Lin

**Affiliations:** 1 Fujian Key Laboratory of Novel Functional Textile Fibers and Materials, Minjiang University, Fuzhou 350108, China; cwlou@asia.edu.tw (C.-W.L.); toyysbk2@yahoo.com.tw (B.-C.S.); 2 Advanced Medical Care and Protection Technology Research Center, College of Textile and Clothing, Qingdao University, Qingdao 266071, China; 3 Department of Bioinformatics and Medical Engineering, Asia University, Taichung 41354, Taiwan; 4 Innovation Platform of Intelligent and Energy-Saving Textiles, School of Textiles, Tianjin Polytechnic University, Tianjin 300387, China; 5 Department of Medical Research, China Medical University Hospital, China Medical University, Taichung 40402, Taiwan; 6 Department of Fiber and Composite Materials, Feng Chia University, Taichung 40768, Taiwan; lai3630@gmail.com; 7 Graduate Institute of Biotechnology and Biomedical Engineering, Central Taiwan University of Science and Technology, Taichung 40601, Taiwan; ctlu@ctust.edu.tw; 8 School of Chinese Medicine, China Medical University, Taichung 40402, Taiwan; 9 Laboratory of Fiber Application and Manufacturing, Department of Fiber and Composite Materials, Feng Chia University, Taichung 40768, Taiwan; 10 Department of Fashion Design, Asia University, Taichung 41354, Taiwan

**Keywords:** polyvinyl alcohol (PVA), nanofiber, Eucalyptus globules essential oil (EGO), *Forcipomyia taiwana* (*F. taiwana*), repellent effectiveness, electrospinning

## Abstract

This study aims to develop nanofibrous membranes where *Eucalyptus globules* oil (EGO) is wrapped in polyvinyl alcohol (PVA). The EGO-based nanofibrous membranes are then evaluated for the protection against Forcipomyia taiwana (*F. taiwana*). In the first stage, the PVA solutions are formulated with different concentrations and are measured for viscosity and electrical conductivity. In the next stage, PVA solution and EGO are blended at different ratios and electrospun into PVA/EGO nanofibrous membranes (i.e., EGO-based repellent). In this study, a PVA concentration of 14 wt% has a positive influence on fiber formation. Furthermore, the finest nanofibers of 291 nm are presented when the voltage is 15 kV. The repellent efficacy can reach 80% in a 60-min release when the repellent is composed of a PVA/oil ratio of 90/10. To sum up, the nanofibrous membranes of essential oil exhibit good repellent efficacy against *F. taiwana* and significant slow-release effect, instead of adversely affecting the cell viability.

## 1. Introduction

Mosquito repellents on the markets are required to provide lasting effective relief and they are thus commonly composed of N,N-diethyl-meta-toluamide (DETT), the most effective substance. Although the constituent DETT is used within the dosage limit, it may still infiltrate the human body and get into the blood as a result of frequent or extensive use of DETT-based repellents. The residual DETT in the human body cannot have complete metabolism until after several months [1]. Some studies stated that the side effects of repellents containing DETT included skin allergy, insomnia, testis atrophy, drop in sperm counts, and damage to the nervous and immune systems [2,3]. The aforementioned disadvantages of DETT mosquito repellents expedite the development of natural plant-based repellents. Unlike chemically synthesized repellents, plant-based repellents cause failures in multiple organs of mosquitos, instead of a specified organ. The active mechanism effectively prevents mosquitos from drug resistance [3]. 

In Taiwan, mountain areas are commonly overlapped with the habitat of *F. taiwana.* Tourists and inhabitants inevitably suffer from *F. taiwana* bites that cause sensitive skin to inflame and itch, and bite victims would even develop allergic reactions and fever. *F. taiwana* is classified as a repulsive pest [4,5,6]. *Eucalyptus globules*, *Melaleuca Alternifolia*, *Cymbopogon*, and *Mentha piperita* are commercially available *F. taiwana* repellency. In particular, *Eucalyptus globules* essential oil (EGO) is biologically active, antimicrobial, and bacteriostatic, and thus can be used as an insect repellent, pesticide, and acaricide [7,8]. Filter papers containing 3 mL/cm^2^ of peppermint essential oil were found fatal to 100%, 90%, and 85% of Culex quinquefasciatus, Anopheles stephensi, and Aedes aegypti, respectively, for the first 24 h [9]. As stated in previous studies, *Eucalyptus globules* contains estragole, terpinolene, 1,4-Hexadiene -methyl-3-(1-methylethylidene), and 1,8-cineole, which are repellent against mosquitos [10,11]. There is little research verifying whether essential oils are effective in the protection against *F. taiwana*. Therefore, this study investigates the repellency of EGO-based nanofibrous membranes. Nanofibrous membranes have been commonly used in research works in the food industry, and are also used to wrap essential oils to obtain the function of drug release. PVA, a hydrolysis product of polyvinyl acetate, is a water-soluble polymer and has good chemical stability, biocompatibility, penetrability, spinnability, and membrane formation [12]. Hence, PVA is used as the carrier of EGO. 

This study designs electrospun mixtures of essential oil and PVA to form EGO repellent and extend the application to compass the protection against *F. taiwana* [13]. The electrospinning technique has come to the fore because the efficient manufacturing process involves simply electrospinning a polymer solution or polymer melt into nanofibrous membranes by means of an electrostatic force. Therefore, electrospinning has many advantages, such as a low production cost, a great diversity of material selections, and high connectivity among fibers, and it can also incorporate functional additives with the resulting nanofibrous membranes. As a result, electrospinning has been commonly used in fields of tissue engineering, wound healing dressings, controlled/sustained release systems, filtration films, and functional textiles. Besides, nanofibrous membranes are featured by high porosity, high surface, ease of operation, and cost-effectiveness. In particular, a high area surface enables the membranes to carry a greater drug loading while a high entrapment efficiency benefits the drug release rate [14,15,16,17].

Moreover, electrospinning is an efficient method to form the PVA/EGO mixtures into nanofibrous membranes [18], involving parameters of the concentration of PVA solution, spinning voltage, the distance between the syringe and the collection plate (i.e., the collection distance), and the velocity of the jet. The viscosity and electrical conductivity of the PVA solution are measured. The surface of the nanofibrous membranes and diameter distribution of nanofibers are examined using a scanning electron microscope (SEM), as is the case with the PVA/EGO nanofibrous membranes that are composed of PVA/EGO ratios of 95/5, 90/10, 85/15, and 80/20. Moreover, the PVA/EGO nanofibrous membranes are also tested in terms of cell viability and repellency using a Y-tube olfactometer. 

## 2. Materials and Methods

### 2.1. Materials

*Eucalyptus globules* essential oil (EGO) was purchased from O’ddenio, Taipei, Taiwan. Polyvinyl alcohol (PVA) powders (Sigma-Aldrich, St. Louis, MO, USA) had a molecular weight of 89,000–98,000. [3-(4,5-Dimethylthiazol-2-yl)-2,5-diphenyltetrazolium bromide] (MTT) powders were dissolved in dimethyl sulfoxide (DMSO). MTT, DMSO, and phosphate buffer solution (PBS) were purchased from Quantum Biotechnology, Taichung, Taiwan. Mouse fibroblast (L929) was purchased from the Bioresource Collection and Research Center, Hsinchu, Taiwan. Cell culture solution containing 90% of Dulbecco’s Modified Eagle Medium (DMEM), 9% horse serum, and 1% of antibiotics was purchased from Quantum Biotechnology, Taiwan.

### 2.2. Preparation of Samples

#### 2.2.1. PVA Nanofibrous Membranes

PVA powders and deionized water were added to a sealed Erlenmeyer flask and mixed for 2 h using a magnetic stirrer, and then cooled for 1 h, thereby forming 12, 14, and 16 wt% PVA solutions. Then, 25 mL of PVA solutions were filled into a #18 stainless steel syringe. The anode and cathode of the spinning voltage were connected to the syringe and the collector respectively. The voltage was between 10 and 20 kV, the flow rate of the PVA solution was 1 mL/h, and the collection distance was 10 cm. The collector was covered with a layer of aluminum foil. The morphology and diameter distribution were observed using an SEM and it was obtained that the optimal concentration of PVA solution was 14 wt%, which was used for the production of PVA/EGO nanofibrous membranes.

#### 2.2.2. PVA/EGO Nanofibrous Membranes

*Eucalyptus globules* essential oil (EGO) and 1 wt% Tween 80 were evenly mixed for 30 min, after which a PVA solution was added and stirred for another 30 min to form PVA/EGO blends at ratios of 95/5, 90/10, and 85/15. The blends were electrospun into PVA/EGO nanofibrous membranes at a voltage of 15 kV. 

### 2.3. Tests

#### 2.3.1. Mechanical Properties

The tests were conducted based on the test standard referred to in a previous study [19]. Made of different manufacturing parameters, nanofibers were collected over a collector plate for a specified length of time. After being removed from the collectors, samples were tested for tensile properties at a tensile rate of 10 mm/min using a universal testing machine (HT-2402, Hung Ta Instrument, Taichung, Taiwan) The distance between the gauge was 10 mm and the samples size was 8 cm × 1 cm. Three samples for each specification were taken for the test in order to test and record the average.

#### 2.3.2. Infrared Moisture Determination Balance

This test was conducted at a temperature of 50 °C for 99 min, after which the release capacity of essential oil was computed using the equation as follows.

release capacity (%)=( Wm−Wt)/Wm×100%

where W_m_ is the weight of the nanofibrous membrane before the test and W_t_ is the weight of the nanofibrous membrane after the test.

#### 2.3.3. Scanning Electron Microscopy (SEM)

Electrospun nanofibrous membranes were observed and photographed using the SEM (Phenom Pure Desktop SEM, Thermo Fisher Scientific, Waltham, MA, USA). Based on the SEM images, 100 nanofibers were observed using Image-Pro Plus version 6.2 (Media Cybernetics, Rockville, MD, USA). The mean of the nanofiber diameter was computed to plot the normal diameter distribution.

#### 2.3.4. MTT Assay

MTT assay was used to measure the cell viability of the PVA/EGO nanofibrous membranes as specified in ISO 10993-5. Fibroblasts at 5 × 10^3^ cells/well in a 96-well culture plate were cultured in a CO_2_ incubator for 24 h and processed in a sterilized laminar flow. The culture medium was removed using a Pasteur pipette, and the sample extract was then added to the plate for the 1 and 3 d culture. Then, the sample extract was removed and an MTT agent was added, after which the plate was kept in the dark for 4 h. The MTT agent was removed, and 70 μL of DMSO was added to serve as the solvent of the crystal. An ELISA reader (Thermo Fisher Scientific, Waltham, MA, USA) was used to measure the absorbance, (i.e., optical density, OD). The ODs were used to compute cell viability, indicating whether the materials have cytotoxicity. The control group was without using the sample extract. Cell Viability was computed using the equation as follows.

Cell Viability=(D of the experimental group/OD of the control group)×100%

#### 2.3.5. Repellent Timeliness Measurement

**Figure 1:**
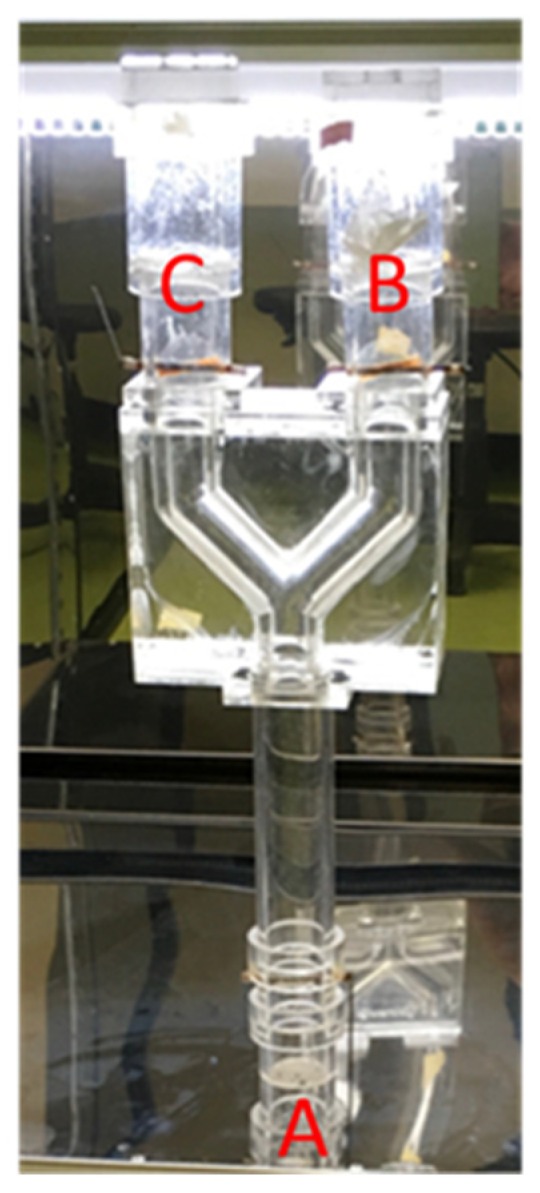
Image of the Y-tube olfactometer.

A total of 20 ± 3 female *F. taiwana* were allocated on the insect end (Figure 1A). An EGO nanofibrous membrane was positioned on the odor end (Figure 1B) of the Y-tube olfactometer and simultaneously a PVA fibrous membrane was placed on the control end (Figure 1C). After 3 min, the EGO membrane was removed for 12 min, and repositioned on the odor end again. This procedure was to prevent *F. taiwana* from being numb with olfaction after they stay in a ligand-odor space. A test cycle lasted 15 min, and the number of F. *taiwana* was recorded for 8 cycles. Namely, the length of a full test was 2 h. A new batch of F. taiwana was used for each test and 3 samples for each specification were used. The repellency rate against *F. taiwana* was computed to have the value in percent using the following equation.

Repellency rate = (1 − (the average data of three samples on end (B)/the total of the *F.taiwana*)) × 100%

## 3. Results and Discussion

### 3.1. Effects of Viscosity and Electrical Conductivity of PVA Solutions on PVA Nanofibrous Membranes

The morphology of PVA nanofibers is dependent on the parameters of electrospinning and PVA solutions. An excessive viscosity or low electrical conductivity has a negative influence on the morphology of nanofibers. Namely, the nanofibers have a greater diameter [20]. Table 1 shows the viscosity and electrical conductivity of the PVA solutions at 12, 14, and 16 wt %. The viscosity and electrical conductivity of the PVA solutions are in proportion to the concentration of the PVA solution. Moreover, regardless of whether it is 10, 15, or 20 kV, the PVA concentration of 16 wt % obtains the greatest thickness, which is ascribed to its high viscosity. A concentration of 16 wt % has the highest viscosity that bonds the nanofibers, which is unfavorable to the expansion and dissociation of the nanofibers. 

**Figure 2:**
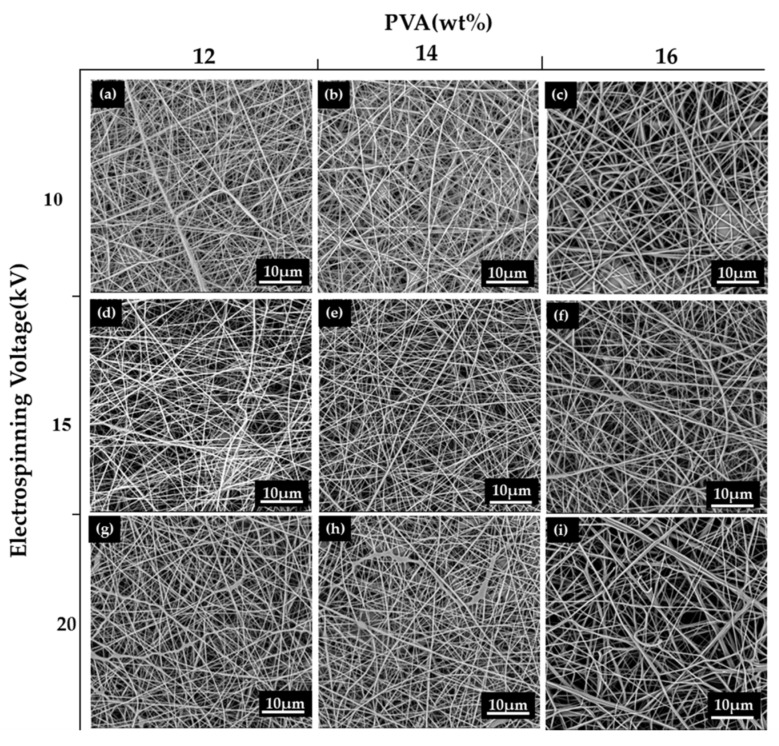
SEM images of PVA nanofibrous membranes with different combinations of concentration of PVA solution (column: 12, 14, and 16 wt%) and spinning voltage (row: 10, 15, and 20 kV).

By contrast, a low concentration of PVA solution cannot keep the PVA jet continual and stabilized, which results in spray with droplets [21,22] that transforms into crimped fibers on the collector. A suitable viscosity that exceeds a critical value prevents the deformation of the jet, which successfully produces uniform nanofibers and then nanofibrous membranes. Figure 2a–i displays the morphology of nanofibers based on different concentrations of PVA solutions. In particular, a concentration of 14 wt% outperforms 12 wt% and 16 wt% in obtaining evenly formed nanofibers. A PVA solution at 12 wt% creates crimped nanofibers, and PVA solution at 16 wt% causes discontinuous or bead-shaped nanofibers. A high concentration renders the reaction between polymers, making the molecular chains entangled and thus forming a jelly-like substance [22]. Moreover, the PVA solution easily forms jelly that clogs the needle and causes instability of the fluid, hindering the electrospinning process. The majority of previous studies employed cross-linking in order to enhance the mechanical strength of PVA nanofibrous membranes. By contrast, cross-linking is absent in this study because it may jeopardize the release of essential oil enwrapped in PVA/EGO nanofibrous membranes. In addition, when practically used, PVA/EGO nanofibrous membranes are situated in a nonwoven bag, which precludes the requirement of mechanical properties. The tensile strength is between 0.23 N and 0.3 N regardless of whether it is a 12 wt%, 14 wt%, or 16 wt% PVA membrane. The effects of enwrapping essential oil on the mechanical properties are discussed in Section 3.3.

### 3.2. Effect of Electrospinning Voltage on Morphology of PVA Nanofibrous Membranes

**Figure 3:**
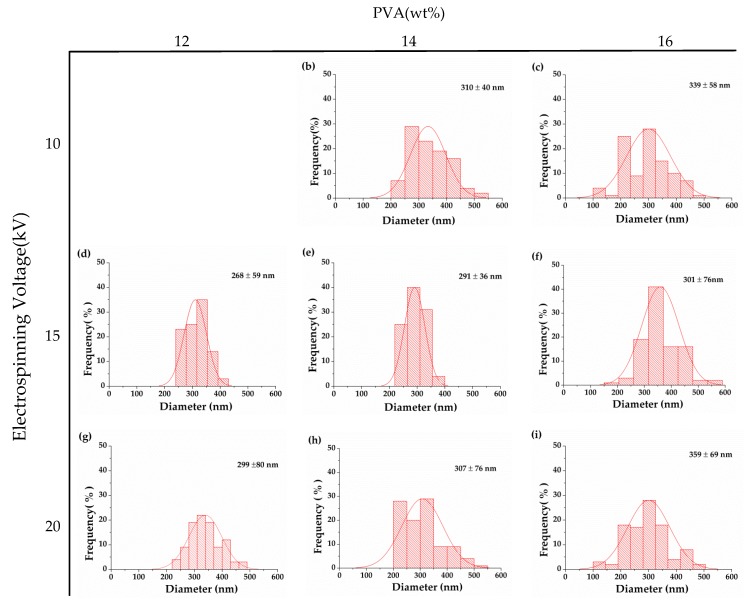
Diameter distribtuion of nanofibrous membranes with different combinations of concentration of PVA solution (column: 12, 14, and 16 wt%) and spinning voltage (row: 10, 15, and 20 kV).

The morphology of nanofibrous membranes correlates closely with the electrospinning parameters. Increasing the collection distance can allot longer evaporation time to the solvent. An excessive collection distance causes an uneven diameter distribution of nanofibers. It also causes the accumulation of spinning solution over the needle, which has an adverse effect on the formation of Taylor cone as well as the evaporation time of the solvent. Based on the observation in our previous study, a collection distance of 10 cm and an optimal jet velocity of 1 mL/h contributed to an optimal morphology of nanofibers [23]. The aforementioned parameters are thus used in this study, and only the voltage is changed to 10, 15, and 20 kV. The voltage is a crucial parameter for the electrospinning process as it influences the shape of droplets, surface electrical load, the withdrawn time of the jet, and expansion of the jet [24]. The test results show that PVA solution can be electrospun into PVA nanofibrous membranes regardless of whether the electrospinning voltage is 10, 15, or 20 kV. When it increases to 15 kV, the evaporation time of the solvent is limited, allowing the jet to be expanded efficiently. Therefore, the nanofibers are evenly formed without beads. When the voltage is 20 kV, a more powerful electric field and a shorter evaporation time make the jet erupting quickly to land on the collection board, resulting in a specified amount of fine nanofibers accompanied by some nanofibers with uneven diameters. Hence, the diameter has a greater range, as shown in Figure 3 [25], and is less ideal. The optimal voltage is proven to be 15 kV, which is in conformity with the finding of the study by Ojha et al. [26]. 

### 3.3. Effect of PVA/EGO Ratio on Morphology of PVA/EGO Nanofibrous Membranes

**Figure 4:**
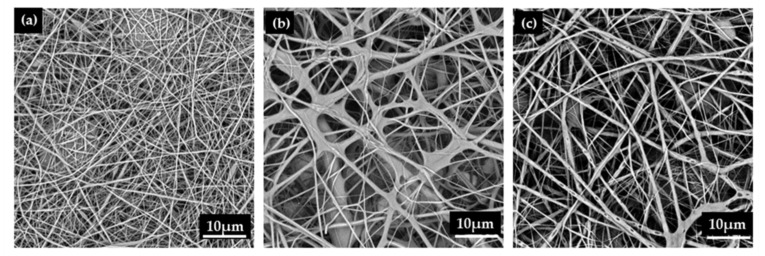
SEM image of PVA/EGO nanofibrous membranes made at a specified spinning voltage of 15 kV. The PVA/EGO ratios are (**a**) 95/5, (**b**) 90/10, and (**c**) 85/15.

**Figure 5:**
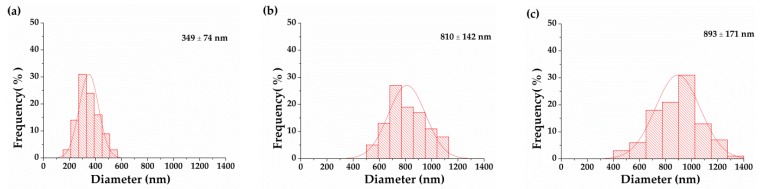
Diameter distribution of PVA/EGO nanofibrous membranes made of a specified spinning voltage of 15 kV. The PVA/EGO ratios are (**a**) 95/5, (**b**) 90/10, and (**c**) 85/15.

Table 2 shows the physical properties and diameter distribution of PVA/EGO nanofibrous membranes. The viscosity of the PVA/EGO blends is proportional to the content of the EGO. An excessive viscosity inhibits the full expansion of the jet during the electrospinning process, and the average diameter is thus higher. The increasing viscosity has a positive influence on the stability of jet dynamics, preventing the jet from dissociating into beads. Although the nanofibers are successfully formed, the average diameter is increased. The PVA/EGO ratio of 80/20 has a viscosity of 2108 cp and an electrical conductivity of 196 µS/cm. Namely, the viscosity is excessive and the electrical conductivity is low. The PVA/EGO blend solidifies in the needle of the syringe and cannot be electrospun into nanofibers, and the 80/20 ratio is thus eliminated from the experiment. Figure 4 and Figure 5 show that with a PVA/oil ratio of 95/5, the nanofiber has a normal distribution of nanofiber diameter between 100 and 400 nm, which demonstrates a relatively greater comparable diameter. Moreover, increasing the oil ratio thickens the nanofiber diameter, but the increase in viscosity is also beneficial for the jet to form the Taylor cone. Regardless of whether the PVA/EGO ratio is 95/5, 90/10, or 85/15, the electrical conductivity of the experimental groups is lower than that of the essential-oil-free nanofibrous membranes (i.e., the control group made of 14 wt% PVA solution as indicated in Figure 2). The electric field of the experimental groups is comparatively lower, which renders the nanofibers with a greater diameter. Figure 4 suggests that under a specified voltage of 15 kV, the diameter of the nanofibers is proportional to the content of the EGO. The diameter increases from 349 nm to 893 nm as a result of increasing EGO from 5 to 15 wt%. Despite the PVA/EGO blending ratio, the tensile strength is between 0.20 N and 0.26 N, which is comparable to the tensile strength of membranes discussed in Section 3.1. This result suggests that enwrapping essential oil does not affect the structure and tensile properties. Moreover, the nanofibrous membranes have enough tensile strength to withstand force when being trimmed. 

### 3.4. Effect of Culture Time on Cell Viability of PVA/EGO Nanofibrous Membranes

**Figure 6:**
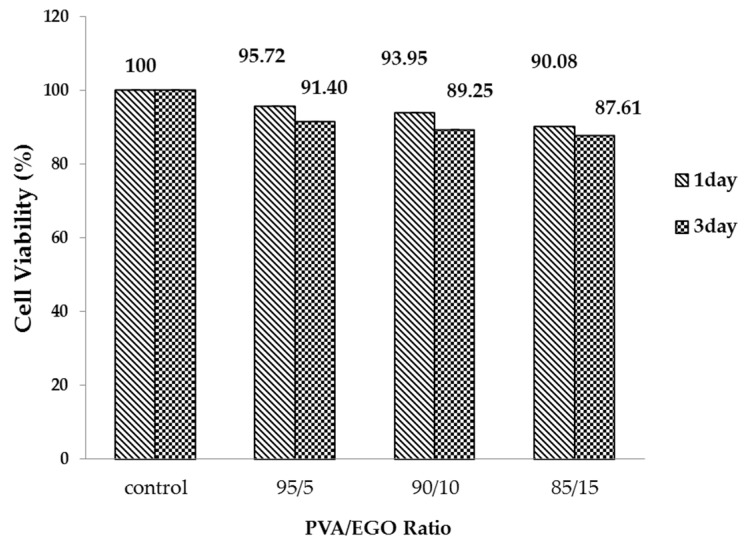
Cell viability of PVA/EGO nanofibrous membranes as related to the culture time (one and three days). The count of fibroblast is 5 × 10^3^ cells/well.

Figure 6 shows that regardless of whether the PVA/EGO ratio is 95/5, 90/10, and 85/15, the cell viability of fibroblasts reaches the standard. For the one-day-culture group, more EGO has a negative influence on the cell viability of fibroblasts. The cell viability is 95%, 93%, and 90% when the PVA/EGO ratio is 95/5, 90/10, and 85/15, suggesting that all of the PVA/EGO nanofibrous membranes have good cell viability. For the three-day-culture group, the cell viability is 91%, 89%, and 87% when the PVA/EGO ratio is 95/5, 90/10, and 85/15. It is clear that cell viability decreases by 3%–4% when the culture time is expanded to three days. This result is due to the fact that the cells have grown and occupied all the space in the culture well in one day. When the culture is expanded to 72 h, there is no space for the newly grown cells, which leads to comparatively lower cell viability.

### 3.5. Effect of PVA/EGO Ratios on Repellent Effectiveness of PVA/EGO Nanofibrous Membranes

**Figure 7:**
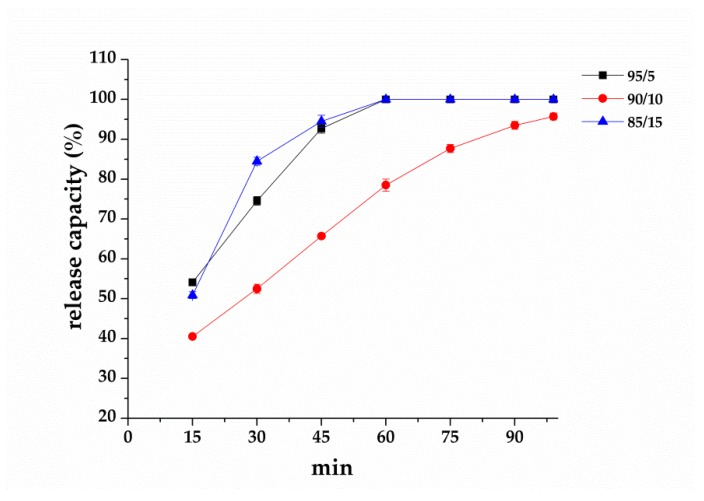
The release capacity (%) of nanofibrous membranes as related to the PVA/EGO ratio.

**Figure 8:**
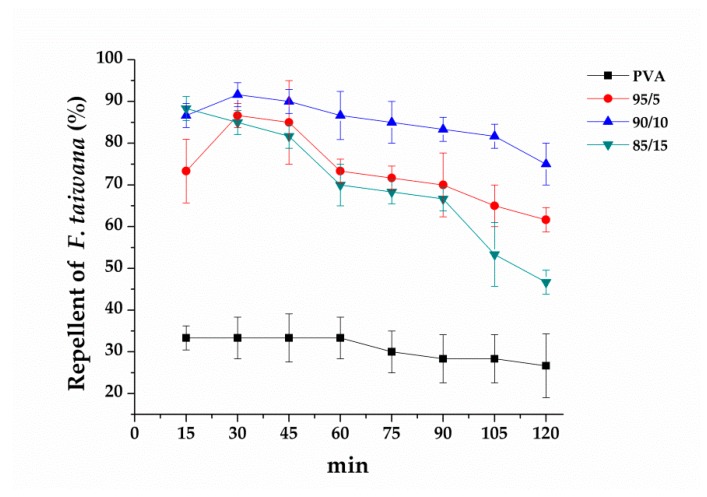
Repellent effectiveness of PVA/EGO nanofibrous membranes as related to the PVA/EGO ratios. The number of *F. taiwana* is 20 ± 3.

In Figure 7, the infrared moisture determination balance test is conducted in order to verify that essential oil is released and whether it shows the repellent effect against F. taiwana. With a PAV/EGO ratio of 95/5 and 85/15, the resulting nanofibrous membranes release the whole essential oil in 90 min. By contrast, the essential oil is not completely released in 99 min from the nanofibrous membranes made with a 90/10 ratio. Because of the absence of valid repellent efficacy against F. taiwana, Y-tube olfactometer is, thus, conducted to make up for it. Figure 8 compares the repellent effectiveness of PVA/EGO nanofibrous membranes in terms of the PVA/EGO ratio and evaporation time. Compared to the repellency of the 95/5, 90/10, and 85/15 groups in the first 15 min, the 95/5 nanofibrous membranes have lower effectiveness of 73%. Compared to the other two groups, the group made with a PVA/EGO ratio of 95/5 yields the finest diameter of 349 nm, as shown in Figure 5. The evaporation of essential oil is more efficient when the fiber diameter is large. Therefore, as for the 95/5 group, the evaporation of essential oil does not generate a saturated gas concentration in the first 15 min, which, in turn, renders this specified group with a lower repellent efficacy. The repellent efficacy is 86.6% for 30 min and 85% for 45 min because the concentration reaches saturation. When exceeding 60 min, the repellent efficacy is reduced from 73% to 61% as a result of a decrease in the released amount of essential oil. When the PVA/EGO ratio is 90/10, the EGO is well wrapped and the repellent effectiveness has a tendency that first increases and then decreases. The nanofibrous membranes give an 85% repellency for the first 15 min and over 90% repellency for the first 30 min. Afterward, the EGO decreases with evaporation time, and the repellency thus eventually decreases. Moreover, the 90/10 group remains a repellent efficacy of 75% in 120 min. By contrast, the repellent effectiveness of 85/15 nanofibrous membranes has a tendency of decreasing with time, which is ascribed to the poor wrapping of EGO. The EGO evaporates so soon, and the 85/15 nanofibrous membranes are short-lived in effectiveness, and the repellent efficacy is reduced from 88% to 46%. In light of the service life, the 90/10 nanofibrous membranes outperform the other two groups, and are proved to be the most effective EGO-based repellent. The 90/10 group gives a 75% repellency lasting 120 min.

## 4. Conclusions

The mixtures composed of PVA solution and essential oil are successfully made into nanofibrous membranes that can repel *F. taiwana* via the electrospinning tsechnique in this study. A raise of PVA concentration from 12 to 14 wt% helps improve the viscosity and electrical conductivity, which, in turn, improves the nanofiber formation. The nanofibers made with a PVA/oil ratio of 95/5 can attain an average diameter of 349 nm and a bead-free morphology. In addition, the results of the cell viability measurement indicate that the nanofibrous membranes composed of PVA/oil with ratios of 95/5, 90/10, and 85/15 do not interfere with the growth of fibroblasts while having cell viability that exceeds 85%, which suggests they do not harm the human cells. In the repellent effectiveness and effective time evaluations, the membranes composed of a PVA/oil ratio of 85/15 exhibit a repellent effectiveness of 45% in two hours, but those composed of a PVA/oil ratio of 90/10 exhibit a repellent effectiveness of 80%. The test results indicate that the proposed PVA/oil nanofibrous membranes have high repellent efficacy against *F. taiwana* and a remarkable slow-release effect, which makes a contribution to the mosquito repellent market.

## Figures and Tables

**Table 1:** Physical properties of polyvinyl alcohol (PVA) solutions and diameter distribution of PVA nanofibrous membranes.

PVA (wt%)	Viscosity (cP)	Electrical Conductivity (µS/cm)	Voltage (kV)	Diameter Distribution (nm)	Average Diameter (nm)	Stress (N)
12	438.6	527	10	223–506	333 ± 63	0.22 ± 0.10
			15	141–482	268 ± 59	0.23 ± 0.05
			20	141–463	299 ± 80	0.26 ± 0.05
14	676	554	10	250–397	310 ± 40	0.23 ± 0.05
			15	235–375	291 ± 36	0.26 ± 0.10
			20	210–538	307 ± 76	0.30 ± 0.10
16	1054	651	10	223–82	339 ± 58	0.23 ± 0.05
			15	141–482	301 ± 76	0.23 ± 0.05
			20	197–559	359 ± 69	0.25 ± 0.10

**Table 2:** Physical properties of PVA/*Eucalyptus globules* oil (EGO) solutions and diameter distribution of PVA/EGO nanofibrous membranes.

PVA (14 wt%) + Essential Oil	Viscosity (cP)	Electrical Conductivity (µS/cm)	Voltage (kV)	Diameter Distribution (nm)	Average Diameter (nm)	Stress (N)
95/5	1405	465	15	197–516	349 ± 74	0.26 ± 0.05
90/10	1586	319	15	530–1080	810 ±142	0.23 ± 0.05
85/15	1847	210	15	442–1353	893 ±171	0.20 ± 0.05
80/20	2108	196	15	-	-	-
